# CLEFMA induces intrinsic and extrinsic apoptotic pathways through ERK1/2 and p38 signalling in uterine cervical cancer cells

**DOI:** 10.1111/jcmm.17671

**Published:** 2023-01-16

**Authors:** Chung‐Yuan Lee, Yi‐Hsuan Hsiao, Pei‐Ni Chen, Heng‐Hsiung Wu, Chih‐Yun Lu, Shun‐Fa Yang, Po‐Hui Wang

**Affiliations:** ^1^ Department of Obstetrics and Gynecology Chiayi Chang Gung Memorial Hospital Chiayi Taiwan; ^2^ Department of Nursing Chang Gung University of Science and Technology Chiayi Taiwan; ^3^ School of Medicine Chung Shan Medical University Taichung Taiwan; ^4^ Department of Obstetrics and Gynecology Changhua Christian Hospital Changhua Taiwan; ^5^ Women's Health Research Laboratory Changhua Christian Hospital Changhua Taiwan; ^6^ Institute of Medicine Chung Shan Medical University Taichung Taiwan; ^7^ Department of Medical Research Chung Shan Medical University Hospital Taichung Taiwan; ^8^ Program for Cancer Biology and Drug Discovery China Medical University Taichung Taiwan; ^9^ Department of Obstetrics and Gynecology Chung Shan Medical University Hospital Taichung Taiwan

**Keywords:** apoptosis, cervical cancer, CLEFMA, ERK, p38

## Abstract

Although concurrent chemoradiotherapy is the cornerstone of treatment for locally advanced or recurrent uterine cervical cancer, treatment fails at a high rate. Therefore, the development of novel targeting agents is critical. This study investigated the action of CLEFMA, a potent, synthetic curcumin derivative, on cervical cancer cells and its mechanism of action. We found that CLEFMA negatively regulated the viability of cervical cancer cells, involving induction of cell apoptosis. Cleaved caspase‐3, cleaved poly(adenosine diphosphate‐ribose) polymerase, cleaved caspase‐8, and cleaved caspase‐9 expression were increased by treatment with CLEFMA. After U0126 (ERK1/2 inhibitor) and SB203580 (p38 inhibitor) were applied as cotreatment with CLEFMA, the expression of cleaved caspase‐8, ‐9, and ‐3 was reduced significantly. In conclusion, CLEFMA activates both extrinsic and intrinsic apoptotic pathways through ERK1/2 and p38 signal transduction in cervical cancer cells.

## 1 INTRODUCTION

Uterine cervical cancer is the fourth most common cancer diagnosis in women and the most lethal malignancy worldwide, and approximately 580,000 patients received diagnoses in 2018.^1^, ^2^, ^3^, ^4^ The annual global incidence of cervical cancer is approximately 14.0 per 100,000 women.^5^ However, current treatments are still lacking; platinum‐based chemotherapy in combination with radiation and concurrent chemoradiation are the preferred treatments for locally advanced or recurrent cervical cancer, but local relapse is often accompanied by distant failure.^6^, ^7^ Therefore, novel agents targeting particular intracellular signalling routes relating to the unique properties of cervical cancer cells are urgently needed.

CLEFMA is a synthetic curcumin analogue (curcuminoid) that was developed as an anticancer agent.^8^, ^9^ Curcumin, a bright yellow, natural polyphenolic compound, is extracted from the rhizomes of the *Curcuma longa* plant.^10^, ^11^, ^12^ It exhibits several pharmacological characteristics, such as anti‐inflammatory, antioxidant, wound‐healing, and antidiabetic properties.^13^, ^14^ Moreover, curcumin may protect against a number of cancers by targeting various biological pathways involved in apoptosis, cell cycle arrest, and activity against several protein kinases.^15^, ^16^, ^17^, ^18^, ^19^ However, the poor water solubility, rapid metabolism, short half‐life, chemical instability, and poor intestinal absorption of curcumin may lead to low plasma levels and thus low bioavailability.^20^ To overcome curcumin's low bioavailability, several approaches, such as adjuvant application and the use of its structural derivatives (e.g. CLEFMA), have been proposed.^21^

CLEFMA possesses superior bioavailability and solubility to those of curcumin; it also exerts antiproliferative effects on cancer cells^8^, ^22^ and is a potential active anticancer compound.^23^, ^24^ Moreover, CLEFMA was demonstrated to have the potential to induce cell death in lung adenocarcinoma cells.^9^ To date, no study has investigated the effect of CLEFMA on cervical cancer cells. Therefore, the present research investigated whether and how CLEFMA induces cell death in cervical cancer cells as well as the underlying mechanisms.

## 2 MATERIALS AND METHODS

### 2.1 Uterine cervical cancer cell culture

HeLa and SiHa cervical cancer cell lines were obtained from the ATCC. Dulbecco's modified Eagle's medium supplemented with 10% fetal bovine serum, 100 ng/ml streptomycin, and 100 ng/ml penicillin was used to culture the cells. For culturing, the HeLa and SiHa cells were maintained at 37°C in an incubator with a 5% CO_2_ humidified atmosphere.^25^

### 2.2 Cell viability

Cell viability was assessed using a 3‐(4,5‐dimethylthiazol‐2‐yl)‐25‐diphenyltetrazolium bromide (MTT; Sigma‐Aldrich) assay. The HeLa cells (7 × 10^4^ cells/well) and SiHa cells (7 × 10^4^ cells/well) were seeded in a 24‐well plate with 100 μl of the culture medium. The medium was removed after 24 h of culturing, and 100 μl of a medium containing 0.5 mg/ml MTT was applied.^26^

### 2.3 Flow cytometry for apoptosis assay

Approximately, 6.5 × 10^5^ HeLa cells and 7.0 × 10^5^ SiHa cells were cultured in one 6‐cm dish in DMEM and treated with CLEFMA for 24 h at one of the following concentrations: 0, 5, 10, 20, or 40 μM. Thereafter, the HeLa and SiHa cells, along with the floating nonviable cells, were harvested through trypsinization. FITC Annexin V Apoptosis Detection Kit I was used to perform an apoptosis assay in accordance with the manufacturer's protocols (BD Biosciences).^27^ Phospholipid phosphatidylserine molecules were translocated from the inner face of the cell membrane to the outer surface of the cell immediately after apoptosis was initiated. Annexin V, a fluorescent conjugated protein exhibiting a high affinity for the phospholipid phosphatidylserine, was applied as a stain to detect apoptosis at an early stage, and propidium iodide (PI) was applied as a stain to detect DNA fragmentation at a later stage. Subsequently, the percentage of cells undergoing apoptosis was determined through flow cytometry.^28^ Quantitative analysis was used to detect early (annexin V positive and PI negative) and late apoptosis (annexin V positive and PI positive) in the HeLa and SiHa cancer cells.

### 2.4 Human apoptosis array

To delineate the underlying mechanisms of induced apoptosis, a Human Apoptosis Array Kit (R&D Systems) was employed to detect protein lysates from cervical cancer cells treated with a vehicle or 20 μM CLEFMA for 24 h, in accordance with the manufacturer's protocols. The kit was used to detect 35 human apoptosis‐related proteins simultaneously.^29^ Proteins were captured on the nitrocellulose membrane, identified with biotinylated detection antibodies, and visualized using chemiluminescent detection reagents.

### 2.5 Protein extraction, detection, and Western blotting

To understand the underlying molecular mechanism, the initiator and effector caspases and signalling pathways were examined using Western blot analysis. We seeded 6.5 × 10^5^ HeLa and 7 × 10^5^ SiHa cervical cancer cells in 6‐cm plates; the cells were cultured for 24 h and subsequently treated with different concentrations (0, 5, 10, 20, or 40 μM) of CLEFMA for 24 h; the total cervical cancer cell lysates were subsequently prepared as previously described.^30^ Western blotting was performed using primary antibodies against caspase‐3, ‐8, and ‐9; cleaved caspase‐3, ‐8, and ‐9; poly(adenosine diphosphate‐ribose) polymerase (PARP); and cleaved PARP. By using the specific antibodies binding the unphosphorylated and phosphorylated forms of the three mitogen‐activated protein kinases (MAPKs), extracellular signal‐regulated kinase (ERK)1/2, c‐Jun N‐terminal kinases (JNK)1/2, and p38, or their inhibitors (U0126, JNK‐IN‐8 and SB203580, respectively), the total cell lysates were obtained from the 6.5 × 10^5^ HeLa and 7.0 × 10^5^ SiHa cells that were cultured for 24 h and then treated or not treated with the three MAPK inhibitors for 2 h and finally treated with various concentrations of CLEFMA. As previously described,^31^ the Western blots were incubated with horseradish peroxidase–conjugated goat anti‐rabbit or anti‐mouse immunoglobulin G for 1 h, and the intensity of each band was measured through densitometry.

### 2.6 Statistical analysis

Statistical analysis of the data was performed using one‐way analysis of variance with a post hoc Scheffe test for comparisons of more than two groups. Each experiment was conducted in triplicate. A two‐sided *p* value of <0.05 was considered statistically different.

## 3 RESULTS

### 3.1 Effects of CLEFMA on HeLa and SiHa cervical cancer cell viability

**FIGURE 1:**
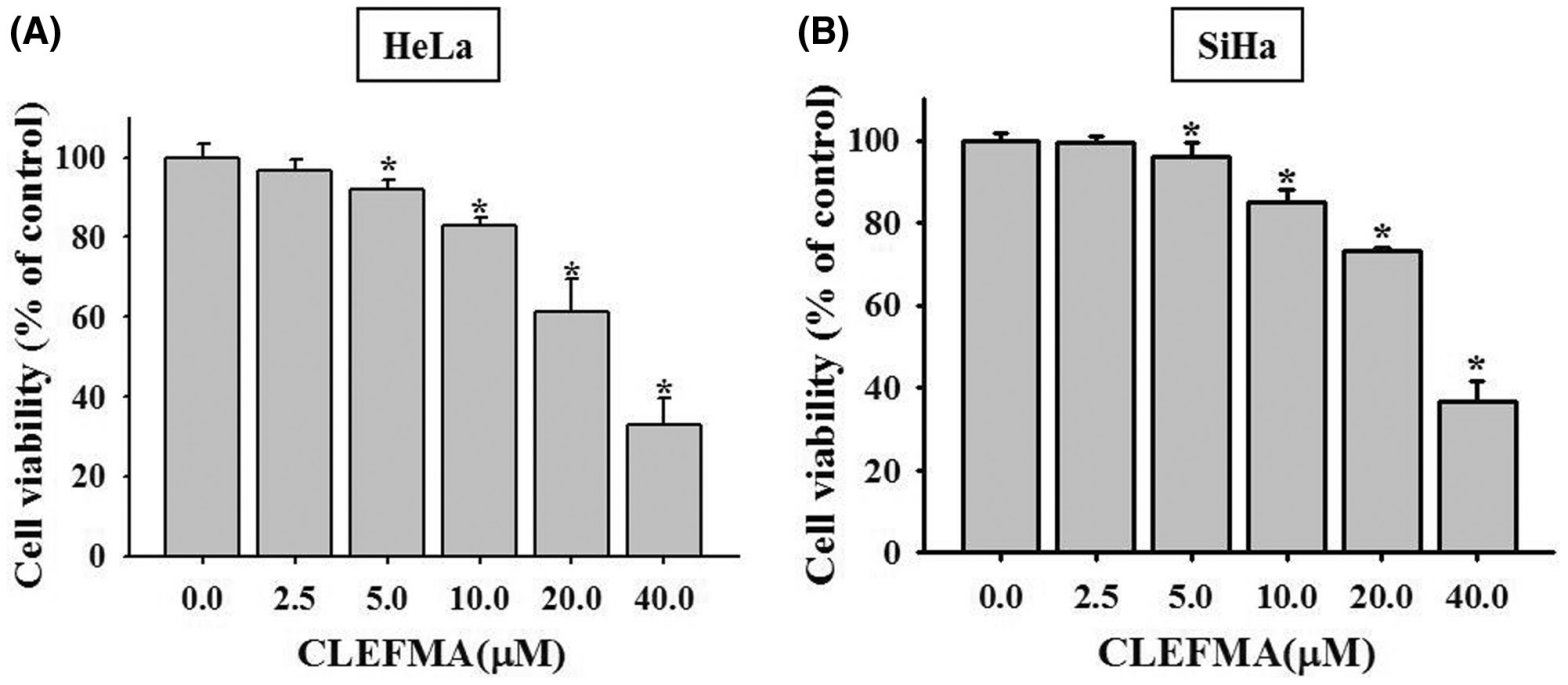
The effects of CLEFMA on cell viabilities of the HeLa and SiHa uterine cervical cancer cell lines using MTT assay. (A) HeLa cells and (B) SiHa cells were treated with different concentrations of CLEFMA (0, 2.5, 5, 10, 20, and 40 μM). Triplet experiments were done. The data were expressed as mean ± SD. Significant differences were defined by one‐way ANOVA with post hoc analysis. *p < 0.05, as compared to controls (no CLEFMA treatment).

The MTT assay revealed that the cell viability of the HeLa cervical cancer cells treated with 2.5, 5, 10, 20, and 40 μM CLEFMA for 24 h was 96%, 91%, 83%, 61%, and 33%, respectively, of that of the control (no CLEFMA) cells. The cell viability of the SiHa cancer cells treated with 2.5, 5, 10, 20 and 40 μM CLEFMA for 24 h was 99%, 96%, 85%, 73%, and 37%, respectively, of that of the control (no CLEFMA) cells. The viability of both the HeLa and SiHa cells treated with 5, 10, 20, or 40 μM CLEFMA was statistically different from that of their respective control (Figure 1A,B).

### 3.2 Flow cytometric analysis of CLEFMA‐induced HeLa and SiHa cervical cancer cell apoptosis

**FIGURE 2:**
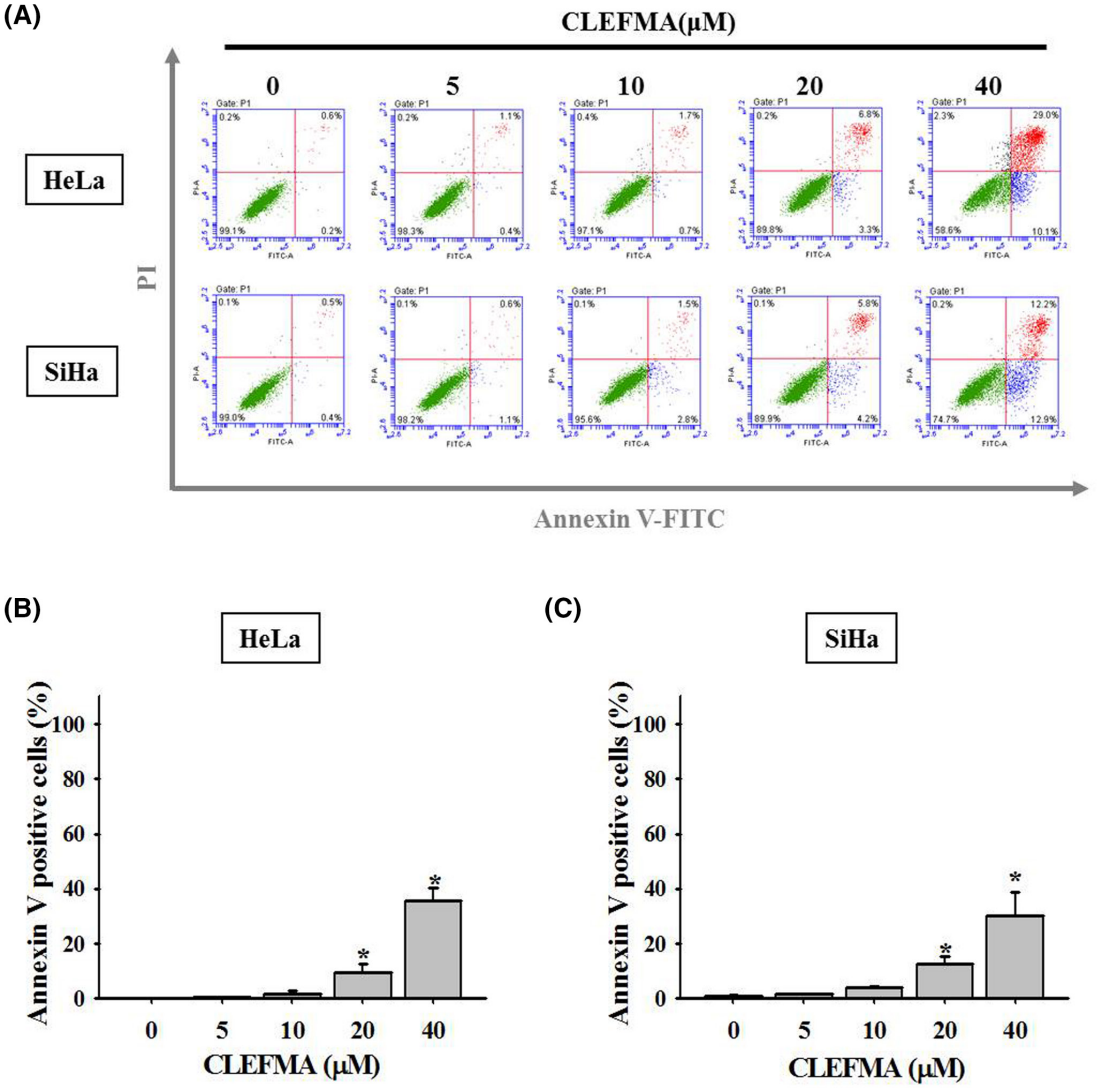
Effects of CLEFMA on the apoptosis of HeLa and SiHa cervical cancer cells. (A) HeLa and SiHa were treated with 0, 5, 10, 20, and 40 μM of CLEFMA and subsequently subjected to flow cytometry after fluorescein isothiocyanate (FITC)‐labelled annexin V and propidium iodide (PI). Both FITC annexin V and PI negative indicate cells that were regarded as visible; FITC annexin V positive and PI negative indicate cells that were in in early apoptosis; both FITC annexin V positive and PI positive indicate cells that were in late apoptosis. The quantitative analysis of early and late apoptosis for (B) HeLa and (C) SiHa cancer cells was summarized as cell apoptosis. Triplet experiments were done. The data were expressed as mean ± SD. Significant differences were defined by one‐way ANOVA with post hoc analysis. *p < 0.05, as compared to controls (no CLEFMA treatment).

To clarify the mechanism underlying the CLEFMA‐induced reduction of HeLa and SiHa cancer cell viability, cell apoptosis was analysed using flow cytometry with fluorescein isothiocyanate–labelled annexin V and PI after treatment with 0, 5, 10, 20, or 40 μM CLEFMA in these cancer cells for 24 h (Figure 2A). Results indicated that the percentage of apoptotic cells exhibited a statistically significant increase in a concentration‐dependent manner in both HeLa and SiHa cells (Figure 2B,C).

### 3.3 CLEFMA increases cleaved caspase‐3 expression in HeLa cervical cancer cells

**FIGURE 3:**
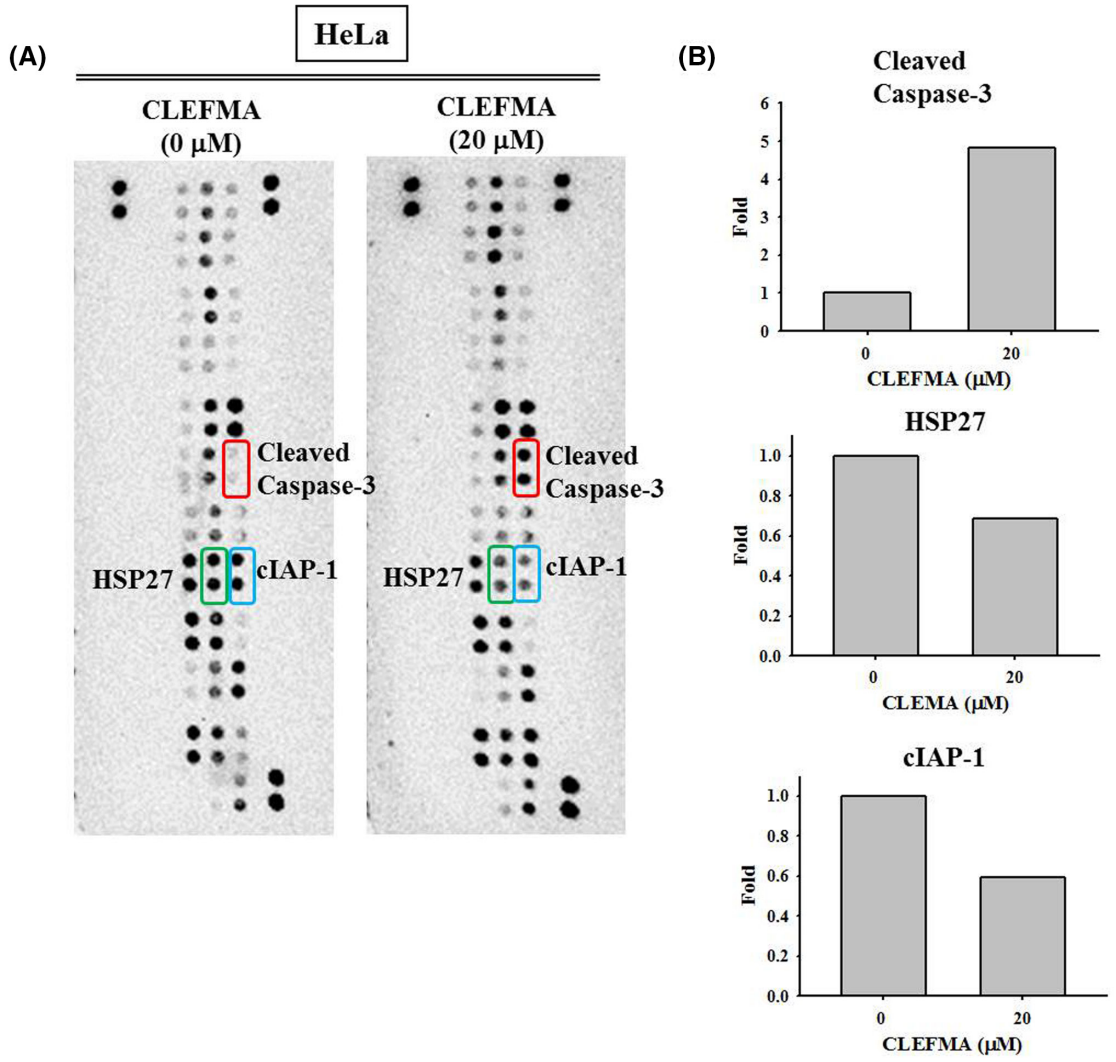
Effect of CLEFMA treatment on the HeLa cervical cancer cells by the human apoptosis array. (A) Expressions of cleaved caspase‐3, heat shock protein 27 (HSP27) and cellular inhibitor of apoptosis protein 1 (cIAP‐1) of total cell lysates from HeLa cells cultured for 24 h and then treated with 20 μM CLEFMA for 24 h by human apoptosis array, as compared to controls (no CLEFMA treatment). (B) The quantitative analysis of apoptosis associated proteins.

To clarify which apoptosis‐related proteins' expression was increased by CLEFMA in the HeLa cervical cancer cells, a human apoptosis array was employed. The results revealed a significant increase in cleaved caspase‐3 as well as decreases in heat shock protein 27 and cellular inhibitor of apoptosis protein 1 in the HeLa cells treated with 20 μM CLEFMA for 24 h (Figure 3A,B).

### 3.4 CLEFMA can trigger the activation of caspase cascade in HeLa and SiHa cervical cancer cells

**FIGURE 4:**
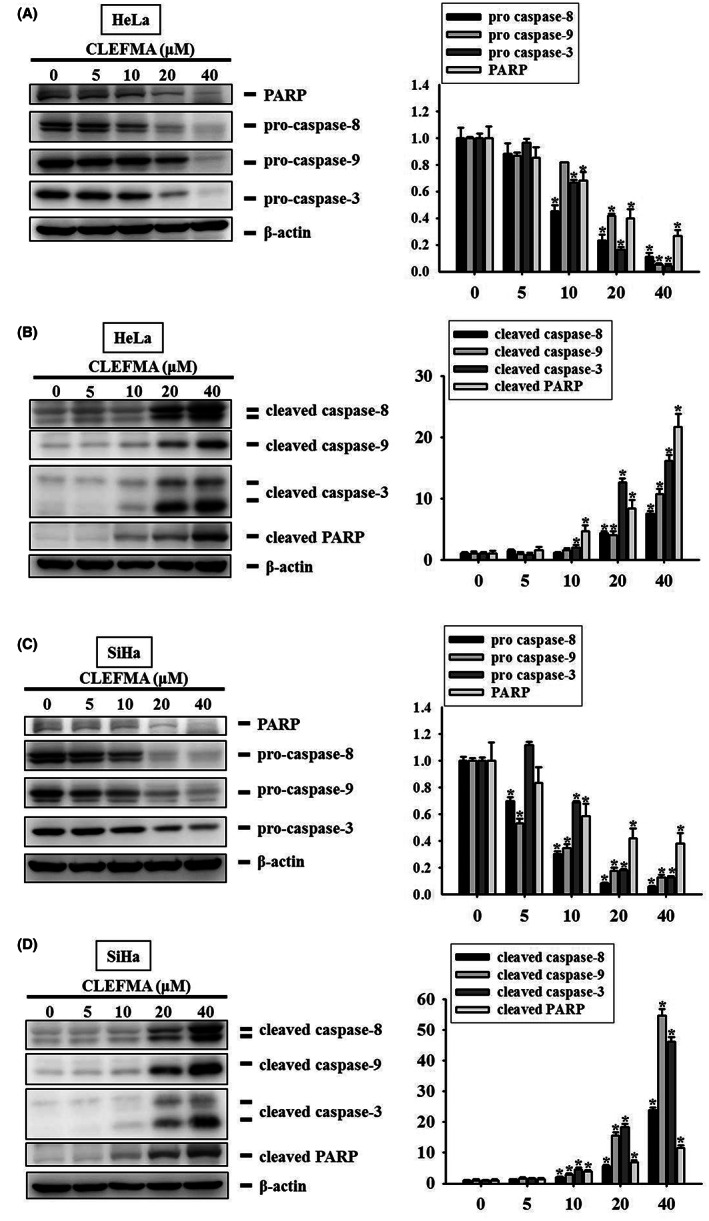
Effects of CLEFMA on the activation of caspase cascade in HeLa and SiHa cervical cancer cells. Less expressions of proform caspases, pro‐caspases‐8, ‐9, and ‐3, as well as poly adenosine diphosphate‐ribose polymerase (PARP) correspond to the higher concentrations of CLEFMA using total cell lysates from (A) HeLa cells and (C) SiHa cells after treatment with 0, 5, 10, 20, and 40 μM of CLEFMA for 24 h by Western blotting. β‐Actin as internal control. The quantitative analysis reveals an inverse proportion relationship. *p < 0.05, as compared to controls (no CLEFMA treatment). Higher expressions of cleaved caspases, caspases‐8, 9, and 3, as well as cleaved PARP correspond to the higher concentrations of CLEFMA using total cell lysates from (B) HeLa cells and (D) SiHa cells by Western blotting. β‐Actin as internal control. Triplet experiments were done. The data were expressed as mean ± SD. The quantitative analysis reveals a direct proportion relationship. *p < 0.05, as compared to controls (no CLEFMA treatment).

To elucidate the effect of CLEFMA on the caspase cascade of the apoptotic signalling pathway, the effector caspase‐3; its upstream initiators, caspase‐8 and caspase‐9; and their respective cleaved forms were detected in HeLa and SiHa cervical cancer cells through Western blotting. After treatment with 0, 5, 10, 20, or 40 μM CLEFMA for 24 h, the expression of the apoptosis‐associated markers procaspase‐8, ‐9, and ‐3 and PARP was inversely proportional to the concentrations of CLEFMA in the HeLa and SiHa cells (Figure 4A,C). By contrast, higher expression of the cleaved forms of caspase‐8, ‐9, and ‐3 and PARP was significantly associated with higher concentrations of CLEFMA in the HeLa and SiHa cells, constituting a directly proportional relationship (Figure 4B,D).

### 3.5 CLEFMA activates extrinsic and intrinsic apoptotic pathways, possibly through MAPK pathways in HeLa and SiHa cervical cancer cells

**FIGURE 5:**
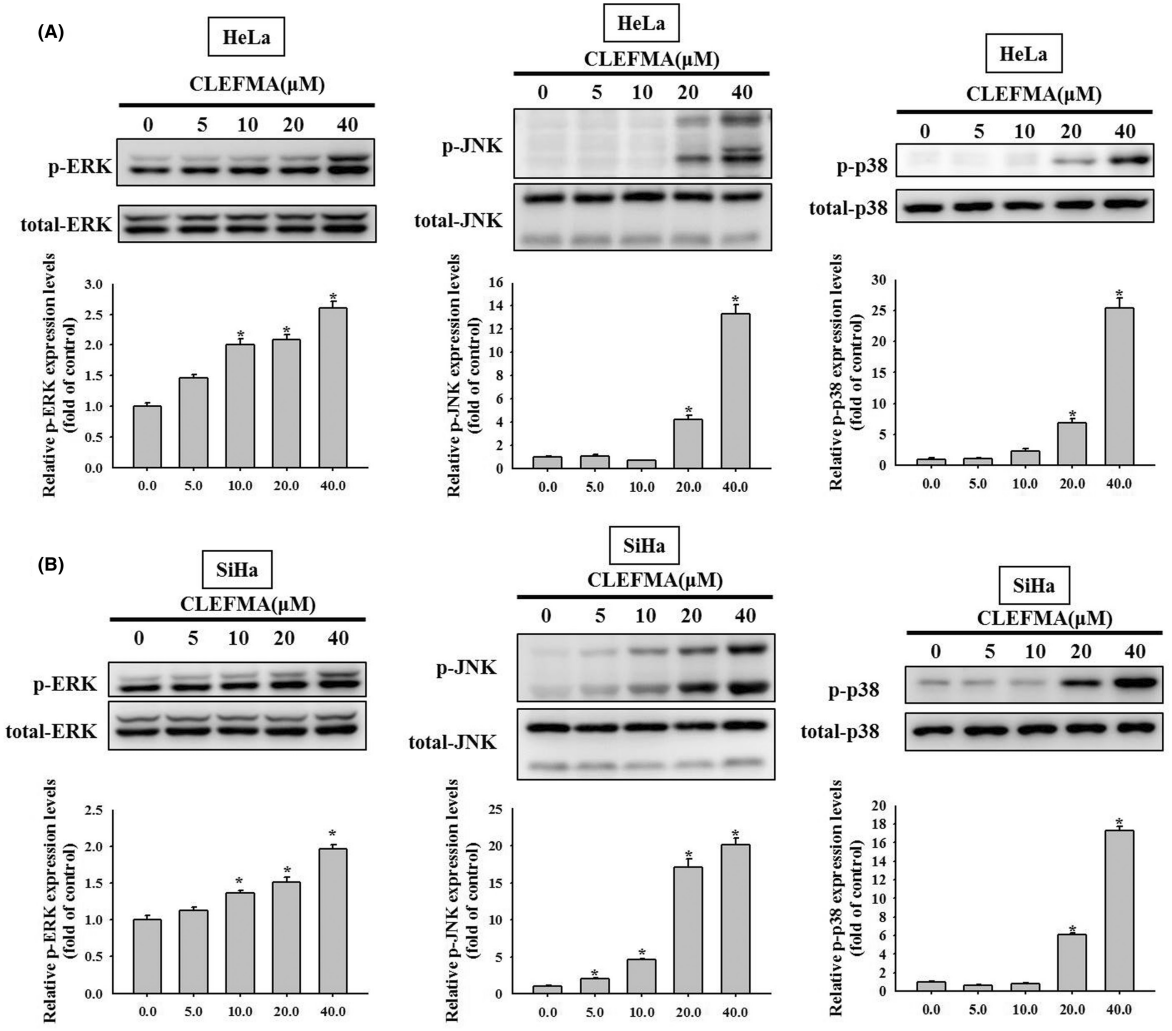
Effects of CLEFMA on the phosphorylation of mitogen‐activated protein kinases (MAPKs), extracellular signal‐regulated kinase (ERK), c‐Jun N‐terminal kinases (JNK) and p38 in HeLa and SiHa cervical cancer cells. Expressions of phosphorylation of ERK, JNK, and p38 using total cell lysates from (A) HeLa and (B) SiHa cells after treatment with 0, 5, 10, 20, and 40 μM of CLEFMA for 6 h by Western blotting. The quantitative analysis for the relative expression levels of phosphorylated ERK, JNK, and p38 were indicated as these phosphorylated proteins divided by their total counterpart proteins. Triplet experiments were done. Data are shown as mean ± SD. Significant differences were defined by one‐way anova with post hoc analysis. *p < 0.05, as compared to controls (no CLEFMA treatment).

Western blot analysis was performed to determine the underlying molecular mechanisms through which MAPK‐related proteins influence the expression of caspase‐8, ‐9, and ‐3 and PARP and thus induce apoptosis in HeLa and SiHa cells. Our experiments revealed that CLEFMA increased the phosphorylation of ERK1/2, JNK1/2, and p38 significantly in a concentration‐dependent manner in HeLa and SiHa cells (Figure 5A,B). These findings suggest that the ERK1/2, JNK1/2, and p38 pathways are involved upstream in the regulation of CLEFMA‐mediated apoptotic signalling through the extrinsic caspase‐8 pathway and intrinsic caspase‐9 pathway and in their downstream effect exerted through caspase‐3 in HeLa and SiHa cervical cancer cells.

**FIGURE 6:**
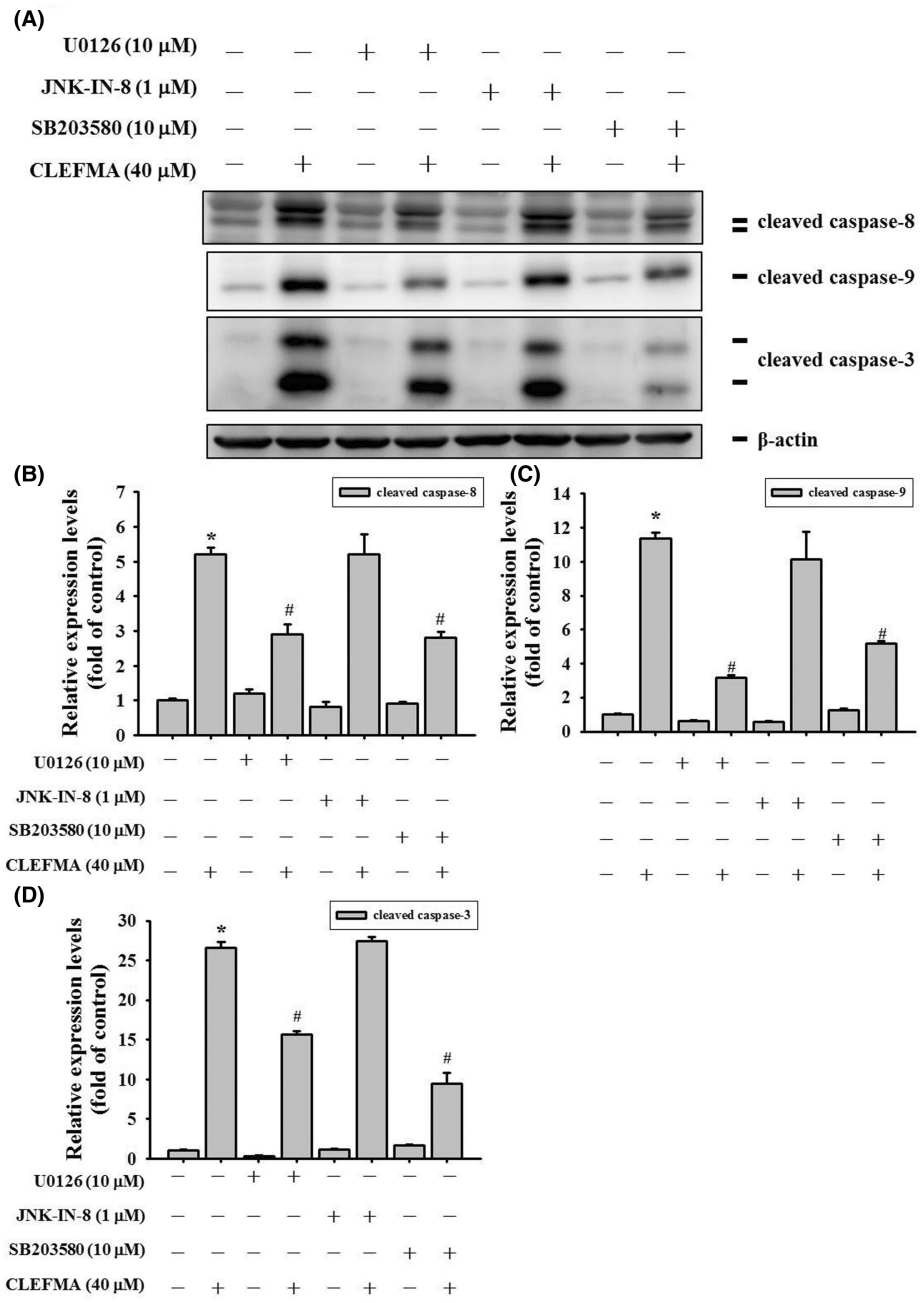
The influences of inhibitors of mitogen‐activated protein kinases (MAPKs) on the expression of activation of cleaved caspases‐8, ‐9, and ‐3 on treatment of CLEFMA in HeLa cervical cancer cells. (A) The expressions of cleaved caspases‐8, ‐9, and ‐3 were assessed based on total cell lysates from HeLa cells after inhibitors of extracellular signal‐regulated kinase (ERK; U0126, 0 or 10 μM), c‐Jun N‐terminal kinases (JNK; JNK‐IN‐8, 0 or 1 μM) or p38 (SB203580, 0 or 10 μM) were given for 2 h and then CLEFMA (0 or 40 μM) for 24 h by Western blotting in HeLa cells. β‐Actin for internal control. Expressions of (B) cleaved caspase‐8 (C) cleaved caspase‐9 and (D) cleaved caspase‐3 in quantitative analysis. Triplet experiments were done. Data are shown as mean ± SD. *p < 0.05, as compared to controls (no drug treatment); #*p* < 0.05, as compared to 40 μM CLEFMA.

To determine which MAPK proteins (i.e. ERK1/2, JNK1/2, or p38) trigger the activation of the caspase cascade, inhibitors of ERK1/2 (U0126, 10 μM), JNK1/2 (JNK‐IN‐8, 1 μM), and p38 (SB203580, 10 μM) were used to assess the proteins' influence on the expression of caspase‐8, ‐9, and ‐3 in combination with 40 μM CLEFMA treatment in HeLa cells (Figure 6A). After the application of the ERK1/2 (U0126) and p38 (SB203580) inhibitors and CLEFMA, the expression of cleaved caspase‐8, ‐9, and ‐3 was reduced significantly, as determined through Western blotting (Figure 6B–D, respectively). However, the JNK inhibitor (JNK‐IN‐8) did not significantly reduce the expression of cleaved caspase‐8, ‐9, or ‐3 after treatment with 40 μM CLEFMA. These results indicate that CLEFMA activates the extrinsic caspase‐8, intrinsic caspase‐9, and caspase‐3 apoptotic pathways mainly through ERK1/2‐ and p38‐mediated pathways.

## 4 DISCUSSION

With advancements in cancer therapy, platinum‐based chemotherapy with irradiation has become the preferred treatment for locally advanced or recurrent cervical cancer. However, treatment still fails a relatively high rate.^7^ Therefore, new modalities, drugs, or agents for the treatment of cervical cancer require development. In addition, the mechanisms or pathways underlying their effects should be delineated. Recently, curcumin has demonstrated potential anticancer effects.

Curcumin has been demonstrated to induce apoptosis in human leukaemia cells, possibly through both intrinsic and extrinsic pathways, which are triggered by MAPK pathways.^32^, ^33^ Moreover, curcumin exhibits antitumor effects on retinoblastoma cells by regulating the JNK and p38 pathways.^34^ However, no research has examined the anticancer effect of curcumin on cervical cancer cells exerted through the inhibition of cell proliferation and the promotion of apoptosis through the ERK, JNK, and p38 signalling pathways.^35^ Despite its efficacy and safety, curcumin has limited bioavailability because it is poorly absorbed and rapidly metabolized.^36^ Accordingly, we chose a structural analogue, CLEFMA—which possesses superior bioavailability and solubility to those of curcumin—as the target for our research.^9^

Apoptotic mechanisms are triggered by extrinsic apoptotic signals, which mainly respond to extracellular stimuli and are associated with death receptors, and by intrinsic apoptotic processes, which are activated by modulators within the cell itself and relate to the mitochondria. The induction and execution of apoptosis signal transduction require the caspase cascade.^37^, ^38^ In this study, a human apoptosis array kit was employed to determine which apoptosis‐related proteins were affected by CLEFMA in cervical cancer cells and revealed significantly increased expression of cleaved caspase‐3. In addition to cleaved caspase‐3 and cleaved PARP, whose expression was significantly increased in HeLa and SiHa cancer cells, the upstream initiators of the extrinsic cleaved caspase‐8 and intrinsic cleaved caspase‐9 pathways were both involved, as determined through Western blotting. This result implies that CLEFMA exerts anticancer effects through extrinsic and intrinsic apoptosis‐related pathways in cervical cancer cells.

CLEFMA was demonstrated to activate both extrinsic and intrinsic apoptotic pathways through the action of MAPKs, including JNK and p38 signalling, in human osteosarcoma cells.^27^ As an adjuvant of anticancer agents, curcumin has been found to induce apoptosis in human breast cancer, melanoma, and oral cancer through MAPK pathways.^39^, ^40^, ^41^, ^42^ GO‐Y078, a synthesized curcumin analogue, can induce cell apoptosis through a variety of mechanisms and has been shown to exert anticancer effects through increased phosphorylation of ERK and JNK in osteosarcoma cells.^43^, ^44^ Although no study has delineated the signalling pathways by which CLEFMA affects cervical cancer cells, we could infer that CLEFMA, a potent curcumin analogue, activates extrinsic and intrinsic apoptotic pathways, possibly through the phosphorylation of ERK, JNK, or p38, in HeLa and SiHa cervical cancer cells. In this study, the expression of phosphorylated ERK, JNK, and p38 progressively increased with the concentration of CLEFMA in HeLa and SiHa cervical cancer cells. To determine the underlying mechanism by which CLEFMA increases the expression of cleaved caspase‐3, ‐8, and ‐9, ERK, JNK, and p38 pathway inhibitors were applied to determine whether they could inhibit the CLEFMA‐induced activation of the caspase cascade. CLEFMA's effect on cleaved caspase‐3, ‐8, and ‐9 was significantly inhibited by cotreatment with CLEFMA and the ERK or p38 inhibitor. Therefore, these findings suggest that CLEFMA activates both extrinsic and intrinsic apoptotic pathways through ERK and p38 signalling, but not through the JNK signalling, in cervical cancer cells.

In conclusion, CLEFMA triggers ERK and p38 signal transduction. CLEFMA activates both the extrinsic apoptotic pathway to increase caspase‐8 expression and the intrinsic apoptotic pathway to increase caspase‐9 expression and subsequently increases the expression of cleaved caspase‐3 and cleaved PARP in cervical cancer cells. By this mechanism of apoptosis induction, CLEFMA reduces the viability in cervical cancer cells.

## AUTHOR CONTRIBUTIONS

**Chung‐Yuan Lee:** Conceptualization (equal); writing – original draft (equal); writing – review and editing (equal). **Yi‐Hsuan Hsiao:** Writing – original draft (equal). **Pei‐Ni Chen:** Methodology (equal). **Heng‐Hsiung Wu:** Methodology (equal). **Chih‐Yun Lu:** Methodology (equal). **Shun‐Fa Yang:** Conceptualization (equal); writing – original draft (equal); writing – review and editing (equal). **Po‐Hui Wang:** Conceptualization (equal); writing – original draft (equal); writing – review and editing (equal).

## FUNDING INFORMATION

This study was supported by research grants from the Chung Shan Medical University Hospital, Taiwan (CSH‐2022‐D‐002). This research was also funded by China Medical University, Taiwan (CMU‐108‐MF‐10).

## CONFLICT OF INTEREST

The authors declare that there is no conflict of interest.

## Data Availability

The data used to support the findings of the present study are available from the corresponding author upon request.

## References

[1] Hsin MC , Hsieh YH , Hsiao YH , Chen PN , Wang PH , Yang SF . Carbonic anhydrase IX promotes human cervical cancer cell motility by regulating PFKFB4 expression. Cancers (Basel). 2021;13(5):1174. 33803236 10.3390/cancers13051174 PMC7967120

[2] Hsin MC , Hsieh YH , Wang PH , Ko JL , Hsin IL , Yang SF . Hispolon suppresses metastasis via autophagic degradation of cathepsin S in cervical cancer cells. Cell Death Dis. 2017;8(10):e3089. 28981104 10.1038/cddis.2017.459 PMC5680581

[3] Cubie HA , Campbell C . Cervical cancer screening – the challenges of complete pathways of care in low‐income countries: focus on Malawi. Womens Health (Lond). 2020;16:1745506520914804. 32364058 10.1177/1745506520914804 PMC7225784

[4] Bray F , Ferlay J , Soerjomataram I , Siegel RL , Torre LA , Jemal A . Global cancer statistics 2018: GLOBOCAN estimates of incidence and mortality worldwide for 36 cancers in 185 countries. CA Cancer J Clin. 2018;68(6):394‐424. 30207593 10.3322/caac.21492

[5] Portnoy A , Clark S , Ozawa S , Jit M . The impact of vaccination on gender equity: conceptual framework and human papillomavirus (HPV) vaccine case study. Int J Equity Health. 2020;19(1):10. 31937328 10.1186/s12939-019-1090-3 PMC6961353

[6] Thigpen T , Shingleton H , Homesley H , Lagasse L , Blessing J . Cis‐platinum in treatment of advanced or recurrent squamous cell carcinoma of the cervix: a phase II study of the gynecologic oncology group. Cancer. 1981;48(4):899‐903. 7196794 10.1002/1097-0142(19810815)48:4<899::aid-cncr2820480406>3.0.co;2-6

[7] Tewari KS , Monk BJ . Evidence‐based treatment paradigms for Management of Invasive Cervical Carcinoma. J Clin Oncol. 2019;37(27):2472‐2489. 31403858 10.1200/JCO.18.02303 PMC7098831

[8] Adams BK , Ferstl EM , Davis MC , et al. Synthesis and biological evaluation of novel curcumin analogs as anti‐cancer and anti‐angiogenesis agents. Bioorg Med Chem. 2004;12(14):3871‐3883. 15210154 10.1016/j.bmc.2004.05.006

[9] Lagisetty P , Vilekar P , Sahoo K , Anant S , Awasthi V . CLEFMA‐an anti‐proliferative curcuminoid from structure‐activity relationship studies on 3,5‐bis(benzylidene)‐4‐piperidones. Bioorg Med Chem. 2010;18(16):6109‐6120. 20638855 10.1016/j.bmc.2010.06.055 PMC2945829

[10] Tomren MA , Masson M , Loftsson T , Tonnesen HH . Studies on curcumin and curcuminoids XXXI. Symmetric and asymmetric curcuminoids: stability, activity and complexation with cyclodextrin. Int J Pharm. 2007;338(1–2):27‐34. 17298869 10.1016/j.ijpharm.2007.01.013

[11] Tomeh MA , Hadianamrei R , Zhao X . A review of curcumin and its derivatives as anticancer agents. Int J Mol Sci. 2019;20(5):1033. 30818786 10.3390/ijms20051033 PMC6429287

[12] Ammon HP , Wahl MA . Pharmacology of Curcuma longa. Planta Med. 1991;57(1):1‐7. 2062949 10.1055/s-2006-960004

[13] Xu XY , Meng X , Li S , Gan RY , Li Y , Li HB . Bioactivity, health benefits, and related molecular mechanisms of curcumin: current Progress, challenges, and perspectives. Nutrients. 2018;10(10):1553. 30347782 10.3390/nu10101553 PMC6213156

[14] Kunnumakkara AB , Bordoloi D , Padmavathi G , et al. Curcumin, the golden nutraceutical: multitargeting for multiple chronic diseases. Br J Pharmacol. 2017;174(11):1325‐1348. 27638428 10.1111/bph.13621 PMC5429333

[15] Kunnumakkara AB , Bordoloi D , Harsha C , Banik K , Gupta SC , Aggarwal BB . Curcumin mediates anticancer effects by modulating multiple cell signaling pathways. Clin Sci (Lond). 2017;131(15):1781‐1799. 28679846 10.1042/CS20160935

[16] Tuorkey MJ . Curcumin a potent cancer preventive agent: mechanisms of cancer cell killing. Interv Med Appl Sci. 2014;6(4):139‐146. 25598986 10.1556/IMAS.6.2014.4.1 PMC4274352

[17] Agarwal A , Kasinathan A , Ganesan R , et al. Curcumin induces apoptosis and cell cycle arrest via the activation of reactive oxygen species‐independent mitochondrial apoptotic pathway in Smad4 and p53 mutated colon adenocarcinoma HT29 cells. Nutr Res. 2018;51:67‐81. 29673545 10.1016/j.nutres.2017.12.011

[18] Talib WH , Al‐Hadid SA , Ali MBW , Al‐Yasari IH , Ali MRA . Role of curcumin in regulating p53 in breast cancer: an overview of the mechanism of action. Breast Cancer (Dove Med Press). 2018;10:207‐217. 30568488 10.2147/BCTT.S167812 PMC6276637

[19] Ismail NI , Othman I , Abas F , Lajis NH , Naidu R . Mechanism of apoptosis induced by curcumin in colorectal cancer. Int J Mol Sci. 2019;20(10):2454. 31108984 10.3390/ijms20102454 PMC6566943

[20] Stohs SJ , Chen O , Ray SD , Ji J , Bucci LR , Preuss HG . Highly bioavailable forms of curcumin and promising avenues for curcumin‐based research and application: a review. Molecules. 2020;25(6):1397. 32204372 10.3390/molecules25061397 PMC7144558

[21] Lu KH , Lu PW , Lin CW , Yang SF . Curcumin in human osteosarcoma: from analogs to carriers. Drug Discov Today. 2022;28:103437. 36372327 10.1016/j.drudis.2022.103437

[22] Sahoo K , Dozmorov MG , Anant S , Awasthi V . The curcuminoid CLEFMA selectively induces cell death in H441 lung adenocarcinoma cells via oxidative stress. Invest New Drugs. 2012;30(2):558‐567. 21181232 10.1007/s10637-010-9610-4 PMC3110543

[23] Raghuvanshi D , Nkepang G , Hussain A , Yari H , Awasthi V . Stability study on an anti‐cancer drug 4‐(3,5‐bis(2‐chlorobenzylidene)‐4‐oxo‐piperidine‐1‐yl)‐4‐oxo‐2‐butenoic acid (CLEFMA) using a stability‐indicating HPLC method. J Pharm Anal. 2017;7(1):1‐9. 29404012 10.1016/j.jpha.2016.09.004 PMC5686864

[24] Chen PN , Lin CW , Yang SF , Chang YC . CLEFMA induces the apoptosis of Oral squamous carcinoma cells through the regulation of the P38/HO‐1 Signalling pathway. Cancers (Basel). 2022;14(22):5519. 36428612 10.3390/cancers14225519 PMC9688613

[25] Hsin MC , Hsiao YH , Chen PN , et al. Dihydromyricetin inhibited migration and invasion by reducing S100A4 expression through ERK1/2/β‐catenin pathway in human cervical cancer cell lines. Int J Mol Sci. 2022;23(23):15106. 36499426 10.3390/ijms232315106 PMC9735508

[26] Hsin IL , Chou YH , Hung WL , Ko JL , Wang PH . The application of arsenic trioxide in ameliorating ABT‐737 target therapy on uterine cervical cancer cells through unique pathways in cell death. Cancers (Basel). 2019;12(1):108. 31906234 10.3390/cancers12010108 PMC7016694

[27] Yang JS , Lin RC , Hsieh YH , et al. CLEFMA activates the extrinsic and intrinsic apoptotic processes through JNK1/2 and p38 pathways in human osteosarcoma cells. Molecules. 2019;24(18):3280. 31505816 10.3390/molecules24183280 PMC6767181

[28] Su CW , Chuang CY , Chen YT , et al. FLLL32 triggers caspase‐mediated apoptotic cell death in human Oral cancer cells by regulating the p38 pathway. Int J Mol Sci. 2021;22(21):11860. 34769290 10.3390/ijms222111860 PMC8584525

[29] Chen CW , Hsieh MJ , Ju PC , et al. Curcumin analog HO‐3867 triggers apoptotic pathways through activating JNK1/2 signalling in human oral squamous cell carcinoma cells. J Cell Mol Med. 2022;26(8):2273‐2284. 35191177 10.1111/jcmm.17248 PMC8995445

[30] Lu KH , Chen PN , Hsieh YH , et al. 3‐Hydroxyflavone inhibits human osteosarcoma U2OS and 143B cells metastasis by affecting EMT and repressing u‐PA/MMP‐2 via FAK‐Src to MEK/ERK and RhoA/MLC2 pathways and reduces 143B tumor growth in vivo. Food Chem Toxicol. 2016;97:177‐186. 27600294 10.1016/j.fct.2016.09.006

[31] Chien MH , Shih PC , Ding YF , et al. Curcumin analog, GO‐Y078, induces HO‐1 transactivation‐mediated apoptotic cell death of oral cancer cells by triggering MAPK pathways and AP‐1 DNA‐binding activity. Expert Opin Ther Targets. 2022;26(4):375‐388. 35361044 10.1080/14728222.2022.2061349

[32] Yang CW , Chang CL , Lee HC , Chi CW , Pan JP , Yang WC . Curcumin induces the apoptosis of human monocytic leukemia THP‐1 cells via the activation of JNK/ERK pathways. BMC Complement Altern Med. 2012;12:22. 22443687 10.1186/1472-6882-12-22 PMC3342909

[33] Zhu GH , Dai HP , Shen Q , Ji O , Zhang Q , Zhai YL . Curcumin induces apoptosis and suppresses invasion through MAPK and MMP signaling in human monocytic leukemia SHI‐1 cells. Pharm Biol. 2016;54(8):1303‐1311. 26134921 10.3109/13880209.2015.1060508

[34] Yu X , Zhong J , Yan L , et al. Curcumin exerts antitumor effects in retinoblastoma cells by regulating the JNK and p38 MAPK pathways. Int J Mol Med. 2016;38(3):861‐868. 27432244 10.3892/ijmm.2016.2676

[35] Cao F , Liu T , Xu Y , Xu D , Feng S . Curcumin inhibits cell proliferation and promotes apoptosis in human osteoclastoma cell through MMP‐9, NF‐kappaB and JNK signaling pathways. Int J Clin Exp Pathol. 2015;8(6):6037‐6045. 26261481 PMC4525815

[36] Anand P , Kunnumakkara AB , Newman RA , Aggarwal BB . Bioavailability of curcumin: problems and promises. Mol Pharm. 2007;4(6):807‐818. 17999464 10.1021/mp700113r

[37] Gazitt Y , Kolaparthi V , Moncada K , Thomas C , Freeman J . Targeted therapy of human osteosarcoma with 17AAG or rapamycin: characterization of induced apoptosis and inhibition of mTOR and Akt/MAPK/Wnt pathways. Int J Oncol. 2009;34(2):551‐561. 19148492

[38] Park H , Bergeron E , Senta H , et al. Sanguinarine induces apoptosis of human osteosarcoma cells through the extrinsic and intrinsic pathways. Biochem Biophys Res Commun. 2010;399(3):446‐451. 20678472 10.1016/j.bbrc.2010.07.114

[39] Chen J , Li L , Su J , Li B , Chen T , Wong YS . Synergistic apoptosis‐inducing effects on A375 human melanoma cells of natural borneol and curcumin. PLoS ONE. 2014;9(6):e101277. 24971451 10.1371/journal.pone.0101277 PMC4074168

[40] Wang K , Zhang C , Bao J , et al. Synergistic chemopreventive effects of curcumin and berberine on human breast cancer cells through induction of apoptosis and autophagic cell death. Sci Rep. 2016;6:26064. 27263652 10.1038/srep26064 PMC4893614

[41] Chen CF , Lu CC , Chiang JH , et al. Synergistic inhibitory effects of cetuximab and curcumin on human cisplatin‐resistant oral cancer CAR cells through intrinsic apoptotic process. Oncol Lett. 2018;16(5):6323‐6330. 30333889 10.3892/ol.2018.9418 PMC6176463

[42] Sun M , Zhang Y , He Y , et al. Green synthesis of carrier‐free curcumin nanodrugs for light‐activated breast cancer photodynamic therapy. Colloids Surf B Biointerfaces. 2019;180:313‐318. 31071571 10.1016/j.colsurfb.2019.04.061

[43] Kudo C , Yamakoshi H , Sato A , et al. Synthesis of 86 species of 1,5‐diaryl‐3‐oxo‐1,4‐pentadienes analogs of curcumin can yield a good lead in vivo. BMC Pharmacol. 2011;11:4. 21619659 10.1186/1471-2210-11-4 PMC3115866

[44] Lu PW , Lin RC , Yang JS , et al. GO‐Y078, a curcumin analog, induces both apoptotic pathways in human osteosarcoma cells via activation of JNK and p38 signaling. Pharmaceuticals (Basel). 2021;14(6):497. 34073773 10.3390/ph14060497 PMC8225057

